# The Latest Crazy Man

**Published:** 1877-09

**Authors:** 


### THE LATEST CRAZY MAN.

Some person has written a book " On the Principles of Organic Life," and what is more remarkable than this, has found a respectable publisher in London—who maintains that all the great virtues to be found in man, are due to the retention in his body of the fæces,

